# Temperature-induced changes in Arabidopsis Rubisco activity and isoform expression

**DOI:** 10.1093/jxb/erac379

**Published:** 2022-09-17

**Authors:** Amanda P Cavanagh, Rebecca Slattery, David S Kubien

**Affiliations:** Department of Biology, University of New Brunswick, Fredericton, NB, E3B 5A3, Canada; Carl R. Woese Institute for Genomic Biology, University of Illinois at Urbana-Champaign, Urbana, IL 61801, USA; School of Life Sciences, University of Essex, Colchester CO4 3SQ, UK; Carl R. Woese Institute for Genomic Biology, University of Illinois at Urbana-Champaign, Urbana, IL 61801, USA; Department of Biology, University of New Brunswick, Fredericton, NB, E3B 5A3, Canada; Western Sydney University, Australia

**Keywords:** Arabidopsis, carboxylation, oxygenation, phenotypic plasticity, photosynthesis, Rubisco

## Abstract

In many plant species, expression of the nuclear encoded Rubisco small subunit (SSu) varies with environmental changes, but the functional role of any changes in expression remains unclear. In this study, we investigated the impact of differential expression of Rubisco SSu isoforms on carbon assimilation in Arabidopsis. Using plants grown at contrasting temperatures (10 °C and 30 °C), we confirm the previously reported temperature response of the four *RbcS* genes and extend this to protein expression, finding that warm-grown plants produce Rubisco containing ~65% SSu-B and cold-grown plants produce Rubisco with ~65% SSu-A as a proportion of the total pool of subunits. We find that these changes in isoform concentration are associated with kinetic changes to Rubisco *in vitro*: warm-grown plants produce a Rubisco having greater CO_2_ affinity (i.e. higher *S*_C/O_ and lower *K*_C_) but lower $k_{{\text{catCO}}_{\text{2}}}$ at warm measurement temperatures. Although warm-grown plants produce 38% less Rubisco than cold-grown plants on a leaf area basis, warm-grown plants can maintain similar rates of photosynthesis to cold-grown plants at ambient CO_2_ and 30 °C, indicating that the carboxylation capacity of warm-grown Rubisco is enhanced at warmer measurement temperatures, and is able to compensate for the lower Rubisco content in warm-grown plants. This association between SSu isoform expression and maintenance of Rubisco activity at high temperature suggests that SSu isoform expression could impact the temperature response of C_3_ photosynthesis.

## Teaser

In Arabidopsis, growth temperature alters the expression of the Rubisco small subunit gene and protein family, which results in the expression of a Rubisco isoform with increased CO_2_specificity at warm growth temperatures.

## Introduction

Ribulose-1,5-bisphosphate carboxylase/oxygenase (Rubisco) catalyses the competing reactions of photosynthetic carboxylation of ribulose-1,5-bisphosphate (RuBP) and photorespiratory RuBP oxygenation. Since it is a dual-substrate enzyme, a defining kinetic feature is the enzyme’s CO_2_/O_2_ specificity factor (*S*_C/O_), which is the ratio of the catalytic efficiencies of its carboxylation reaction $\left(k_{{\text{catCO}}_{\text{2}}}/K_{\text{C}}\right)$ and oxygenation reaction $\left(k_{{\text{catO}}_{\text{2}}}/K_{\text{O}}\right)$, where *k*_cat_ is the enzyme’s turnover rate and *K*_C_ and *K*_O_ are the apparent Michaelis–Menten constant for each substrate. Rubisco is approximately 2000 times more ­specific for CO_2_ than O_2_, but because the latter is some 500 times more abundant in today’s atmosphere, RuBP oxygenation is a significant limitation to carbon gain, and plays an important role in the photosynthetic temperature response (Sage and Kubien, 2007; Moore *et al.*, 2021).

As temperatures rise, the rate of both Rubisco carboxylation and oxygenation increase, as increased temperatures lower the activation barrier for the addition of both substrates (Sage, 2002; Galmés *et al.*, 2015). However, the carboxylation:oxygenation ratio decreases as temperatures rise, both because the ratio of [CO_2_]:[O_2_] in solution and Rubisco *S*_C/O_ decline. Consequently, photorespiration increases with rising temperature, limiting potential growth and productivity (Walker *et al.*, 2016), making photorespiration an important bioengineering target for crop improvement and resilience (South *et al.*, 2019; Roell *et al.*, 2021; Cavanagh *et al.*, 2022). The increased photorespiratory pressure of hot, dry, arid environments has driven the evolution of carbon concentrating mechanisms, including C_4_ and C_2_ photosynthesis (Lundgren and Christin, 2016; Sage *et al.*, 2018). Within C_3_ plants, there is evidence that warm growth temperatures can also alter Rubisco *S*_C/O_ or thermotolerance, improving Rubisco performance and carbon assimilation at warm growth temperatures (Yamori *et al.*, 2006; Cavanagh and Kubien, 2014). Yamori *et al.* (2006) found that Rubisco from cold-grown (15/10 °C) *Spinacia oleracea* (spinach) is 5% more specific for CO_2_ than O_2_ at 10 °C than Rubisco from warm-grown (30/25 °C) plants, but when measured at 35 °C, the warm-grown Rubisco is 11% more specific. Theoretically, the growth temperature-mediated changes in spinach Rubisco *S*_C/O_ may convey a carbon-gain advantage during acclimation (Cavanagh and Kubien, 2014). The mechanism behind these kinetic changes is not apparent, but likely involve some structural changes to the enzyme.

In land plants and green algae, Rubisco is a hexadecameric complex of eight large and eight small polypeptide subunits (referred to as an L8S8 enzyme form). The Rubisco catalytic site is located at the interface of two 55 kDa large subunits (LSu), which are encoded by the *rbcL* gene in the chloroplast genome. The 15 kDa small subunits (SSu) are produced in the cytosol by nuclear-encoded *RbcS* genes and imported to the chloroplast. Though not directly involved in catalysis, the SSus are required for maximum Rubisco activity (Andrews, 1988). Further, Rubisco containing SSu mutations, or hybrid Rubiscos with native LSus and foreign SSus, often have altered holoenzyme stability and kinetic properties (Spreitzer *et al.*, 2005; Ishikawa *et al.*, 2011; Sharwood *et al.*, 2016; Martin-Avila *et al.*, 2020).

The nuclear encoded *RbcS* genes have more sequence diversity than the chloroplastic *rbcL*, and in many species are present in multigene families ranging from two copies in *Chlamydomonas* to 22 copies in several *Flaveria* species (Spreitzer, 2003; Kapralov *et al.*, 2010). Within the multigene family, differential *RbcS* gene expression has been reported in response to environmental and development cues in a range of species (Wanner and Gruissem, 1991; Dedonder *et al.*, 1993; Meier *et al.*, 1995; Cheng *et al.*, 1998; Eilenberg *et al.*, 1998; Yoon *et al.*, 2001; Suzuki *et al.*, 2009). Arabidopsis has four *RbcS* genes that show differential expression in response to environmental parameters such as light, CO_2_, and temperature (Dedonder *et al.*, 1993; Cheng *et al.*, 1998; Yoon *et al.*, 2001; Sawchuk *et al.*, 2008). However, the impact of these gene-expression changes on Rubisco structure and performance remains unclear.

Unlike the widespread interspecific variation in *RbcS* sequence and copy number, the SSu amino acid sequences within a single species are fairly well conserved. For example, in *Solanum tuberosum* (potato) SSu isoforms have 93% sequence identity. However, Martin-Avila *et al.* (2020) engineered tobacco plants to express L8S8 Rubisco containing both the potato LSu and a single potato SSu isoform, and found that Rubisco containing only the SSu-3 isoform had a 9% increase in *k*_catCO2_ coupled with impaired protein synthesis, which translated into changes in growth and photosynthetic carbon gain. Thus, it is clear that even small differences in amino acid sequence can impart physiologically relevant changes in Rubisco’s kinetic phenotype. By contrast, the four Arabidopsis SSu peptides share a 97% sequence similarity, and *RbcS* mutant lines expressing different SSu complements do not show altered photosynthetic phenotypes in today’s atmosphere (Izumi *et al.*, 2012; Khumsupan *et al.*, 2020), nor are the kinetic properties of these Rubiscos different at 25 °C (Atkinson *et al.*, 2017). However, the potential kinetic contribution of alternative SSu isoforms under variable environmental conditions has not been fully explored (Cavanagh and Kubien, 2014; Cavanagh, 2020).

In Arabidopsis, gene-specific expression within the *RbcS* family varies with both temperature and CO_2_ conditions; growth at low temperatures or high CO_2_ increases *RbcS1A* expression, while growth at high temperatures or ambient CO_2_ increases *RbcS3B* expression (Cheng *et al.*, 1998; Yoon *et al.*, 2001), but changes in *RbcS* gene expression have not been correlated with changes in Rubisco performance. In this work, we aim to determine: (i) if the differences in *RbcS* expression correlate with Rubisco protein expression under different growth temperature regimes; (ii) whether this difference corresponds with changes in Rubisco kinetics *in vitro*; and finally (iii) whether these *in vitro* differences impact the photosynthetic phenotype *in vivo.*

## Materials and methods

### Plant growth and sampling

Arabidopsis (Col-0) seeds were stratified for 3 d at 4 °C on Promix (Plant Products, Brampton, Canada), transferred to a growth chamber (E-15 Conviron, Winnipeg, Manitoba, Canada) and grown under photoperiod conditions of 10 h light/14 h dark, 20/18 °C, 300 µmol m^−2^ s^−1^ photosynthetic photon flux density, under ambient (400–450 µmol mol^−1^) and elevated (1000 µmol mol^−1^) CO_2_ levels. After 1 week, plants were transferred to either a warm (30/27 °C), a cold (10/8 °C), or a moderate (20/18 °C) growth temperature treatment. Temperature and CO_2_ treatments were applied as a factorial design over two growth chambers, ­swapping chambers account for any chamber effects (Supplementary Fig. S1). Leaf temperatures (measured with thermocouples) during the ‘day’ were 8.8, 19.2, and 27.4 °C at 10, 20, and 30 °C air temperatures, respectively. Plants were watered to excess every 2 d (at 30 °C), and every 4 d (10 °C and 20 °C) with a modified Hoagland’s nutrient solution. To normalize nitrogen application to a rate of 160 mmol N week^−1^, 30 °C plants with a solution containing 8 mM total N, and 20 °C- and 10 °C-grown plants were watered with a 16 mM total N solution. Leaf tissue for RNA extraction and biochemical analyses was sampled from the youngest fully expanded leaf of the fourth whorl of 5- to 6-week-old plants (growth stage 3.70–3.90). Leaf discs were obtained 6–7 h after simulated dawn, flash frozen in liquid nitrogen, and stored at −80 °C until extraction.

### Total plant RNA extraction and cDNA synthesis

Total RNA was extracted from flash-frozen leaf material using the RNeasy Plant RNA extraction kit (Qiagen, Hilden, Germany) according to the manufacturer’s instructions. Concentration and relative purity were measured using a spectrophotometer (NanoVue, GE Healthcare Life Sciences), and RNA integrity was confirmed visually on an agarose gel stained with SYBRSafe (Thermo Fisher Scientific). One microgram of RNA was reverse transcribed into cDNA using the QuantiTect Reverse Transcription Kit (Qiagen). Gene expression was measured via quantitative PCR in 20 µl reaction volumes containing 10 µl KAPA SYBR FAST qPCR Master Mix (2X) (KAPA Biosystems, Woburn, MA, USA), 200 nM of gene-specific forward and reverse primers, and 1 µl of cDNA. Real time quantification of amplicons was performed using a Rotor-Gene 6000 thermal cycler (Corbett Life Sciences, Sydney, NSW, Australia), using the following standard thermal profile for all reactions: 95 °C for 2 min, followed by 40 cycles of 95 °C for 3 s, 58 °C for 20 s, and 72 °C for 2 s. Primers for *rbcL* were 5ʹ-GTGTTGGGTTCAAAGCTGGT-3ʹ and 5ʹ-CATCGGTCCACACAGTTGTC-3ʹ (Supplementary Table S1). Primers flanked regions in the 3ʹ UTR of each *RbcS* gene, using a common reverse primer for both *RbcS1A* and *RbcS3B* genes. Product specificity was confirmed visually by band presence/absence following agarose electrophoresis, and via dissociation curves with single peaks obtained for each reaction product.

To extrapolate absolute expression of mRNA copy numbers from the RT-qPCR assay, gene-specific standard curves were prepared in triplicate from known copy numbers of plasmids containing the target sequence of each primer set. All plasmid dilutions used for the standard curves amplified consistently, indicating a linear amplification range of at least six orders of magnitude, from 10^2^ to 10^8^ copies µl^−1^. Expression was normalized to a reference gene (*AT1G13320*), which is consistently expressed across changes in growth temperature in Arabidopsis (Czechowski *et al.*, 2005). Controls containing no template cDNA (NTC) and total RNA (no-RT) were included in every run, and they were not amplified below PCR cycles nor did they cross the quantification cycle threshold (*C*_q_) for samples included in the analysis.

### Rubisco immunoblotting

To examine changes in SSu composition, a quantitative immunoblotting procedure modified from Yamori and von Caemmerer (2011) was used, using a dilution of chromatographically purified spinach Rubisco as a standard. Three micrograms of total Arabidopsis soluble protein and 0.25–2 µg of spinach Rubisco standard in Laemmli buffer (2% SDS, 10% glycerol, 60 mM Tris-Cl pH 6.8) were denatured at 100 °C for 3 min and separated with SDS-PAGE on a 22.4 cm 4–20% acrylamide gel. Proteins were visualized with Coomassie Brilliant Blue (R-250), or transferred to polyvinylidene difluoride membranes pre-wetted in methanol and equilibrated in transfer buffer (2.5 mM Tris, 19.2 mM glycine, 20% methanol, pH 8.6) for 60 min at 100 V. Immediately after transfer, membranes were blocked with 3% non-fat milk (Carnation) in 20 mM Tris, 150 mM NaCl, and 0.1% (v/v) Tween-20 (TBST) for 1 h at room temperature with agitation, or 4 °C overnight. The blot was probed with a polyclonal primary anti-SSu rabbit antibody (Agrisera AS07259, Vännäs, Sweden) diluted 1:5000 in 1% milk in TBST, and an alkaline phosphatase-conjugated goat-anti-rabbit secondary antibody was used (Sigma-Aldrich A3687, St Louis, MO, USA) to develop blots using an alkaline phosphatase Immun-Blot kit (Bio-Rad Laboratories, Mississauga ON, Canada). Protein levels on immunoblots were quantified via densiometry (Quantity One Software, Bio-Rad).

### Rubisco extraction and quantification

Rubisco was prepared from frozen ground leaf tissue (1.1 cm^2^ disks) in a Tenbroek glass-in-glass homogenizer containing 3 ml of ice-cold extraction buffer (100 mM HEPES pH 7.6, 2 mM Na-EDTA, 5 mM MgCl_2_, 5 mM dithiothreitol (DTT), 10 mg ml^−1^ polyvinyl polypyrrolidone, 2% (v/v) Tween-80, 2 mM NaH_2_PO_4_, 12 mM amino-*n*-caproic acid, and 2 mM benzamidine) and 50 µl Protease Inhibitor Cocktail (P9599, Sigma). The chlorophyll content of the leaf homogenate was determined spectrophotometrically after extraction in 80% buffered acetone (Porra *et al.*, 1989). This homogenate was centrifuged at 16 000 *g* at 4 °C for 60 s, and total soluble protein (Bradford, 1976) and *k*_catCO2_ measured from this freshly extracted supernatant. For Michaelis–Menten constants and *S*_C/O_ measurements, the supernatant was desalted (Econo-Pac 10DG desalting column, Bio-Rad) and further concentrated using a spin column as described in Boyd *et al.* (2019) (Amicon 50K spin filters, Millipore, Billerica, MA, USA). Fresh and concentrated aliquots were incubated with 20 mM MgCl_2_ and 10 mM NaHCO_3_ at 30 °C for 20 min to fully carbamylate Rubisco. Rubisco catalytic sites in the carbamylated extract were determined using a [^14^C]carboxy-arabinitol bisphosphate binding assay, with a specific activity of 3.1 kBq nmol^−1^ Rubisco, assuming eight binding sites per Rubisco (Kubien *et al.*, 2011). Rubisco *k*_catCO2_*, K*_C_, *K*_O_, and *S*_C/O_ were determined via radiolabel techniques exactly as described in Boyd *et al.* (2019).

### Gas exchange measurements

The CO_2_ response of net CO_2_ assimilation (e.g. *A*_N_–*C*_i_ curves) was measured using an open gas exchange system (Li-6400, LI-COR Inc., Lincoln, NE, USA). To minimize the impact of developmental stage on measurements, plants were sampled during vegetative growth (i.e. before flower formation; Flexas *et al.*, 2007). Gas exchange was measured in five plants from each growth temperature at 10 °C and 30 °C. Measurements at 10 °C were obtained in a temperature-controlled growth chamber (Conviron PGC-20, Controlled Environments Ltd). The leaf cuvette was set at a reference CO_2_ (*C*_a_) of 400 µmol mol^−1^ and saturating light (500 or 750 µmol m^−2^ s^−1^ at 10 °C and 30 °C, respectively) to reach steady state and then *C*_a_ was decreased in a stepwise fashion from 400, 200, 150, 100, 75, 50, 300, 400, 400, 500, 750, 1200 (both), 1800 (30 °C only) µmol mol^−1^ CO_2_, and measurements made as soon as stability was achieved at each CO_2_ level, typically within 1–3 min. Respiration (*R*_d_) was measured following a dark-acclimation period of 10 min at 400 µmol mol^−1^ CO_2_.

Estimates of mesophyll conductance (*g*_m_), *V*_cmax_, and *J*_max_ were obtained following the curve-fitting method of Ethier and Livingston (2004) using *in vitro* Rubisco parameter values and temperature responses of the Michaelis–Menten constant for CO_2_ (*K*_C_) and oxygen (*K*_O_) and the CO_2_ compensation point in the absence of day respiration (Γ*) calculated from the Rubisco specificity factor (*S*_C/O_) from warm- and cold-grown Arabidopsis. The transition point between the Rubisco and RuBP regeneration-limiting portions of the curve was determined as the *C*_i_ that minimized the difference between *g*_m_ estimates from both processes (Ethier *et al.*, 2006).

At low CO_2_ concentrations the photosynthetic rate of a C_3_ plant is determined by the capacity of Rubisco carboxylation, as the substrate concentration is generally below the Michaelis–Menten constant for the enzyme. To asses Rubisco carboxylation *in vivo*, the initial slopes of *A*_N_–*C*_C_ curves from warm- and cold-grown plants measured at 10 °C and 30 °C were compared to assess Rubisco carboxylation activity *in vivo*. An *in vivo* estimate for the Rubisco Michaelis–Menten constant for CO_2_ in the presence of oxygen (*K*_C21%O2_) was then determined by regressing individual *A*–chloroplastic CO_2_ concentration (*C*_c_) curves against a Michaelis–Menten equation of the form:


$$\frac{A}{\left[\text{Rubisco}\right]}=\frac{(C_{c}-\Gamma *)kcat\text{CO}2}{C_{c}+K_{C21\%\text{O}}{}_{2}}$$ (1)

where *K*_C21%O2_=*K*_C_(1+*O*/*K*_O_), Γ* is the compensation point, and [Rubisco] is the Rubisco site concentration.

### Statistics and modeling

The temperature response of Rubisco parameters was determined by calculating in Sigmaplot (Systat Software) the activation energy from an Arrhenius relationship of the form:


$$Parameter\;\left(T\right)=\text{Parameter}\left({\text{25 }}^{{}^{\circ}}\text{C}\right)\times \text{exp}\left[\left(T-\text{25}\right)E_{\text{a}}/\text{298}R\left(\text{273}+T\right)\right]$$ (2)

where *R* is the universal gas constant (8.314 J K^−1^ mol^−1^), *E*_a_ is the activation energy (kJ mol^−1^) and *T* is the assay temperature (°C). Differences in gene expression, protein expression and Rubisco content, and the activation energy and value at 25 °C for each Rubisco parameter were compared using ANOVA and post-hoc Tukey’s test, considering differences to be significant at *P*<0.05. A two-way ANOVA (with growth and measurement temperature as main effects) was used to compare *in vivo* photosynthetic parameters, and *K*_C21%O2_. All statistical analysis was performed in R (R Core Team, 2020).

The CO_2_ response of Rubisco carboxylation (*V*_C_) was modelled according to the equation:


$$V_{\text{c}}=\left(V_{\text{cmax}}\times C\right)/\left(C+K_{\text{c21}\%\text{O2}}\right)$$ (3)

using *k*_catCO2_ as an estimate for *V*_cmax_, and calculating *K*_C21%O2_ from the *in vitro* determined values of *K*_C_ and *K*_O_ reported in Table 2.

**Table 2.** Rubisco biochemical parameters at 25 °C, and the corresponding activation energy of the temperature response, from plants grown at three air temperatures.

	Growth temperature		
Parameter	10 °C	20 °C	30 °C
*k*_catCO2_ (s^−1^)	3.31 (0.09)^a^	3.11 (0.08)^b^	2.78 (0.10)^c^
*E*_a_ (kJ mol^−1^)	54.2 (0.83)^a^	52.7 (11.9)^a^	48.7 (1.7)^a^
*S*_C/O_ (M M^−1^)	75.1 (1.0)^a^	76.9 (1.0)^ab^	78.0 (0.83)^b^
*E*_a_ (kJ mol^−1^)	−23.7 (1.0)^a^	−22.3 (0.37)^ab^	−19.9 (0.48)^b^
*K*_C_(μM)	11.8 (0.49)^a^	11.5 (0.52)^a^	11.4 (0.70)^a^
*E*_a_ (kJ mol^−1^)	54.8 (4.4)^a^	55.9 (5.6)^a^	40.4 (3.3)^b^
*K*_O_ (μM)	275.6 (7.5)^a^	276.1 (13.3)^a^	259.7 (35.7)^a^
*E*_a_ (kJ mol^−1^)	5.47 (0.94)^a^	8.81 (4.56)^a^	5.94 (1.94)^a^

Data represent the mean and SE of 5–7 replicates. Significant differences, as determined by ANOVA (*P*<0.05), are indicated by different letters.

## Results

### Growth temperature alters Rubisco subunit gene and protein expression

**Fig. 1.**
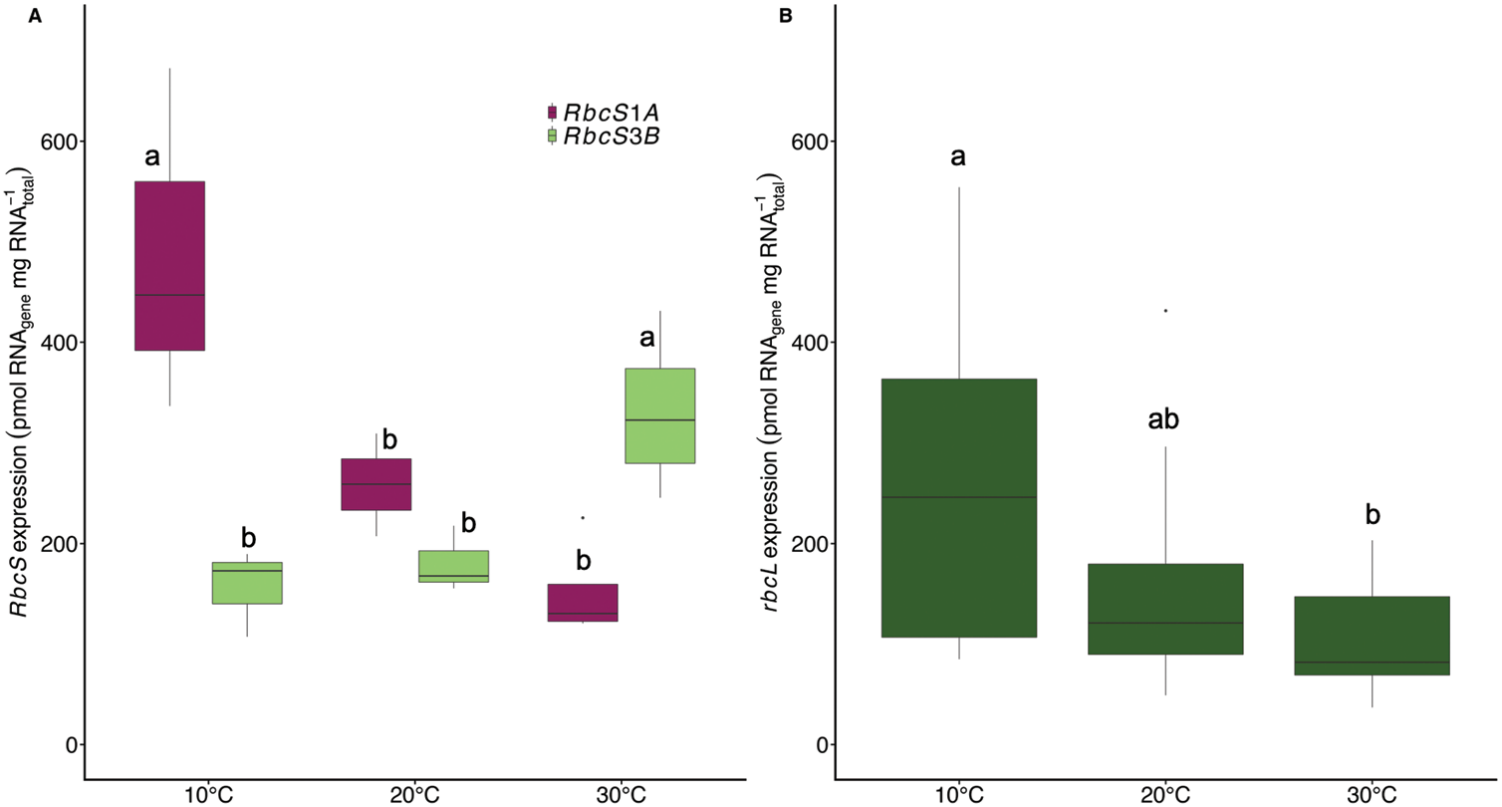
Expression of Rubisco-encoding genes at ambient CO_2_ conditions. Response of the two dominant members of the *RbcS* gene family (A) and *rbcL* gene expression (B) to changes in growth temperature. Gene specific expression is reported as the fraction of total RNA concentration. *n*=6–7. Maximum and minimum values are depicted by the bars, the box signifies the upper and lower quartiles, and the median is represented by a short black line within each box. Different letters represent significant differences in the mean of each treatment at *P*<0.05 (two-way ANOVA and post-hoc Tukey’s HSD test). Responses of all *RbcS* genes can be found in Supplementary Fig. S2.

**Fig. 2.**
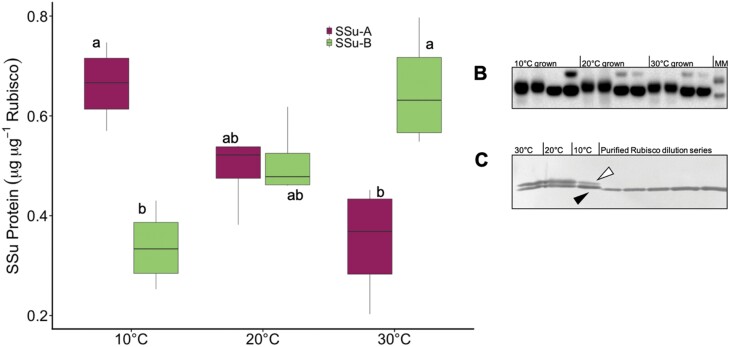
Rubisco protein expression responses to growth temperature. Rubisco SSu isoform expression (A) based on total soluble protein extracted from Arabidopsis leaves and detected by immunoblotting with an anti-LSu (B) or anti SSu (C) antibody. The first two lanes of each temperature treatment in (B) were extracted with a DTT-free extraction buffer, while the second and third lanes contained 5 mM DTT. In samples extracted with 5 mM DTT, trace amounts (~5%) of LSu peptides migrate in a larger molecular mass complex, consistent with an LSu–SSu cross-linked product. MM, molecular mass ladder. White arrowheads in (C) indicate the SSu-B family proteins, while black arrowheads indicate the SSu-1A protein. The purified Rubisco dilution series from left to right is 0.25, 0.5, 0.9, 1.8, and 2 μg. Each lane in the gel represents one extraction from an individual plant. Rubisco SSu protein expression (A) is reported as the fraction of Rubisco SSu present in either band 1 (SSu-1A, grey) or band 2 (SSu-B family, white) on immunoblots (*n*=6). Maximum and minimum values are depicted by the bars, the box signifies the upper and lower quartiles, and the median is represented by a short black line within each box. Different letters represent significant differences in the mean of each treatment at *P*<0.05 (one way ANOVA and post-hoc Tukey’s HSD test).

Growth temperature alters the expression of both *rbcL* and *RbcS* transcripts (Fig. 1). Warm-grown plants produced 38% less Rubisco (measured on a leaf area basis) than plants grown at 10 °C (Table 1, *P*<0.05). This decrease in Rubisco content was coupled with a 56% reduction in *rbcL* transcript expression (Fig. 1B, *P*<0.05), and was not associated with proteolytic degradation visualized as banding on an immunoblot (Fig. 2B). The mRNA levels of *RbcS1A* and *RbcS3B*, and the ratio between them, varied with changes in growth temperature (*RbcS1A*: *P*=0.028; *RbcS3B*: *P*=0.0001 Fig. 1), but not CO_2_ (*RbcS1A*: *P*=0.5014; *RbcS3B*: *P*=0.732; Supplementary Fig. S1). Transcript levels of the *RbcS1A* gene declined as growth temperature increased (Fig. 1A), while those of *RbcS3B* mRNA were higher at 30 °C versus 10 °C (Fig. 1A). As a result, at low growth temperatures *RbcS1A* dominated *RbcS* gene expression, but its mRNA levels declined by 46% when grown at 20 °C and 67% at 30 °C (Fig. 1A). By contrast, mRNA levels of *RbcS3B* were 119% higher at 30 °C than at 10 °C (Fig. 1A). At 20 °C, levels of *RbcS3B* and *RbcS1A* did not differ from expression levels in cold-grown plants at 400 or 1000 µmol mol^−1^ CO_2_, but were 62% lower or 63% greater than expression levels of the respective genes at 30 °C (Fig. 1A; Supplementary Fig. S1). Growth temperature had no effect on the expression of *RbcS*1B and *RbcS*2B (Supplementary Fig. S2), and the expression of these two genes was significantly lower than that of *RbcS1A* and *RbcS3B*.

**Table 1.** Physiological parameters from plants grown at three temperatures.

	Growth temperature		
Parameter	10 °C	20 °C	30 °C
Rubisco content (μmol_sites_ m^−2^)	14.4 (0.83)^a^	10.4 (0.32)^b^	8.9 (0.61)^c^
Total soluble protein (g m^−2^)	4.88 (0.9)^a^	3.39 (0.4)^ab^	2.49 (0.5)^b^
Chlorophyll (mmol m^−2^)	0.852 (0.14)^a^	0.656 (0.064)^a^	0.525 (0.074)^a^
Chlorophyll *a*/*b* (mol mol^−1^)	2.92 (0.19)^a^	2.78 (0.16)^a^	2.33 (0.27)^a^
Rubisco/chlorophyll (mmol mol^−1^)	24.6 (1.2)^a^	19.9 (1.7)^a^	19.8 (1.1)^a^
Specific leaf area (m^2^ kg^−1^)	3.24 (0.42)^a^	1.60 (0.11)^b^	1.24 (0.11)^b^

Chlorophyll content, Rubisco content and total soluble protein were determined on the same extract used for measurements of *k*_catCO2_. Specific leaf area was determined on leaf punches after 5 d of oven drying. Values are means of 5–9 replicates (±SE). Significant differences as determined by ANOVA with *P*<0.05 are indicated by different letters.

Two mature SSu proteins with different molecular mass (predicted to be 14.7 kDa and 14.8 kDa) were separate by SDS-PAGE (Fig. 2B). This separation was similar to previous reports of Arabidopsis SSu protein migration (Getzoff *et al.*, 1998; Izumi *et al.*, 2012). Following the nomenclature of Izumi *et al.* (2012) the larger protein is denoted as SSu-B (where the *RbcS3B* gene product is the predominant component, and has the N-terminal sequence XKVWPP; Izumi *et al.*, 2012) and the smaller protein as SSu-A (which has the N-terminal sequence XQVWPP; Izumi *et al.*, 2012). In cold-grown plants, SSu-A represents 62% of total SSu protein, but only 22% of total SSu protein in warm-grown plants (Fig. 2A, *P*<0.05). By contrast, SSu-B represents 38% and 78% of total SSu protein from 10 °C- and 30 °C-grown plants, respectively (Fig. 2A, *P*<0.05).

### Plants from contrasting growth temperatures have kinetically distinct Rubiscos

**Fig. 3.**
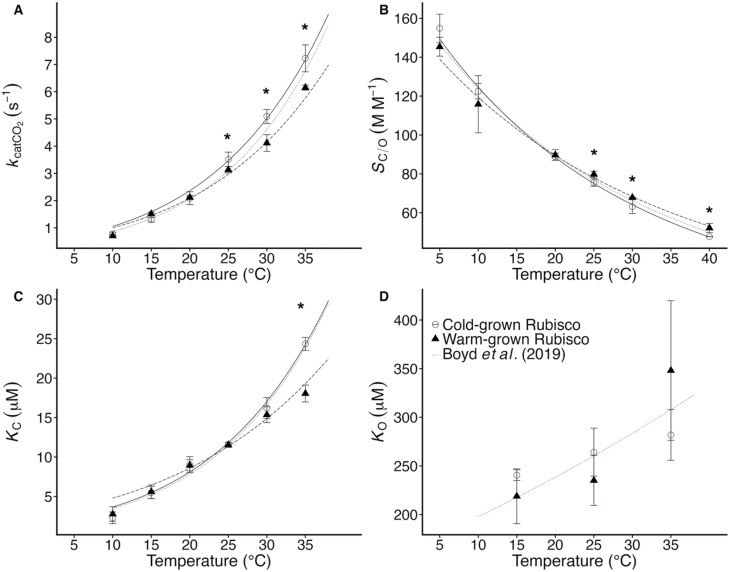
Temperature response of Rubisco kinetics from cold- and warm-grown Arabidopsis. Temperature dependence of the *in vitro* Rubisco *k*_catCO2_ (A), *S*_C/O_ (B), *K*_C_ (C), and *K*_O_ (D) from warm-grown (triangles) and cold-grown (open circles) Arabidopsis. Parameters were determined by assaying activated Rubisco extracts at the indicated temperatures. Data represent means ±SE of 4–7 replicates. The data for *k*_catCO2_, *K*_C_, and *S*_C/O_ were fitted to: (Parameter(*T*)=Parameter(25 °C)exp[(*T*−25)*E*_a_/(298*R*(273+*T*))], where *R* is the universal gas constant (8.314 J K^−1^ mol^−1^), and *T* is the temperature in °C. Lines represent the temperature response of cold (solid) and warm (dashed) Rubiscos, and the dotted line is the temperature response from 20 °C-grown Arabidopsis Rubisco as reported in Boyd *et al.*, 2019.

Rubisco from plants grown at 20 °C represent an approximately intermediate phenotype in all kinetic parameters assayed (Table 2). Measured at 25 °C, Rubisco’s turnover rate (*k*_catCO2_) was 3.31 s^−1^ and 2.78 s^−1^ from plants grown at 10 °C and 30 °C, respectively (*P*<0.05). The activation energy (*E*_a_) of *k*_catCO2_ did not vary with growth temperature (Table 2), but the parameter was greater than warm-grown Rubisco at measurement temperatures above 25 °C (Fig. 3A).

At 25 °C, the CO_2_/O_2_ specificity (*S*_C/O_) of cold-grown Rubisco was 75.1 M M^−1^, while in warm-grown Rubisco it was 78.0 M M^−1^ (*P*<0.05). The *S*_C/O_ of cold-grown Rubisco declined more rapidly with temperature than warm-grown Rubisco, with an apparent *E*_a_ of −23.7 kJ mol^−1^ reported for Rubisco from 10 °C plants and −19.9 kJ mol^−1^ for 30 °C plants (Table 2; *P*<0.05). At all measurement temperatures above 20 °C, *S*_C/O_ was greater in warm- versus cold-grown Rubisco (Fig. 3B; Supplementary Fig. S3).

At 25 °C, the Michaelis–Menten constant for CO_2_ (*K*_C_) was 11.6 μM and did not vary with growth temperature (Table 2). The activation energy (*E*_a_) of cold-grown Rubisco *K*_C_ was 54.8 kJ mol^−1^, while warm-grown Rubisco *K*_C_ had an *E*_a_ of 40.5 kJ mol^−1^ (Table 2, *P*<0.05). As a result of this change in temperature response, at 35 °C cold-grown Rubisco *K*_C_ was 26% greater than warm-grown Rubisco (Fig. 3C). The Michaelis–Menten constant for O_2_ (*K*_O_) did not vary with growth temperature (Table 2; Fig. 3D) and all measurements fell within the 95% confidence intervals of 20 °C-grown Rubisco (Fig. 3D).

### Effects of growth temperature on leaf characteristics

There was no significant difference between the amount of chlorophyll or the ratio of chlorophyll *a*/*b* between plants grown at different temperatures (Table 1). The ratio of Rubisco/chlorophyll appeared 24% larger in cold-grown plants than warm-grown plants (*P*=0.07; Table 1), but this difference was due to the increased production of Rubisco in cold-grown plants, and not a decrease in chlorophyll content. Warm-grown plants also produced less soluble protein than cold-grown plants (2.5 versus 4.9 mg cm^−2^), and had 56% lower specific leaf area (Table 1).

### Growth temperature impacts on plant gas-exchange: rates, parameters, and limitations

Despite the reduction in Rubisco content in warm-grown plants compared with cold-grown plants (Table 1), estimates of *V*_cmax_ varied with measurement temperature (*P*<0.001), but not growth temperature (*P*=0.22). Estimates of the maximal rate of electron transport (*J*_max_) varied with both growth (*P*=0.0004) and measurement temperature (*P=*0.0005) such that warm-grown plants had a lower *J*_max_ at both growth temperatures (Table 3).

**Table 3.** Photosynthetic parameters from cold (10 °C) or warm (30 °C)-grown plants.

	Cold-grown Arabidopsis		Warm-grown Arabidopsis	
	10 °C	30 °C	10 °C	30 °C
*A*_400_ (μmol CO_2_ m^−2^ s^−1^)	19.2 (1.4) ^a^	13.2 (2.1) ^bc^	11.8 (0.51) ^b^	14.7 (0.91) ^c^
*g*_s_ (mol H_2_O m^−2^ s^−1^)	0.26 (0.03) ^ab^	0.20 (0.02) ^b^	0.44 (0.06) ^a^	0.37 (0.11) ^ab^
*K*_C21%O2_ (μM)	17.7 (4.6) ^a^	54.9 (8.5) ^b^	15.6 (2.2)^a^	31.7 (4.9)^c^
*J*_max_ (μmol e^−^ m^−2^ s^−1^)	133.1 (5.5) ^a^	190.6 (16.7) ^b^	74.1 (8.2) ^c^	138.8 (7.7) ^a^
*V*_cmax_ (μmol CO_2_ m^−2^ s^−1^)	55.8 (1.8) ^a^	108.1 (7.2) ^b^	40.1 (5.0) ^a^	100.9 (10.6) ^b^
*R*_d_ (μmol CO_2_ m^−2^ s^−1^)	1.1 (1.4)^a^	3.6 (0.6)^b^	1.1 (0.2)^a^	2.6 (0.3)^c^
*g*_m_ (mol CO_2_ m^−2^ s^−1^)	0.114 (0.01)^a^	0.142 (0.03)^a^	0.131 (0.03)^a^	0.204 (0.07)^a^

The maximum carboxylation rate (*V*_cmax_) and electron transport rate (*J*_max_) were estimated from CO_2_ assimilation curves measured at 10 °C and 30 °C using the temperature responses of cold- and warm-grown Arabidopsis Rubisco Michaelis–Menten constants for CO_2_ and O_2_ in Table 1, and the *in vivo K*_C21%O2_ was estimated from fitting *A*–*C*_c_ curves to the Michaelis–Menten form of the Farquhar model for RuBP saturated photosynthesis. *R*_d_ was measured in a dark-acclimated leaf during gas-exchange measurements. Mean values (*n*=4–7) and SE are reported, and significant differences between values are indicated by different letters according to a two-way ANOVA with *P*<0.05.

**Fig. 4.**
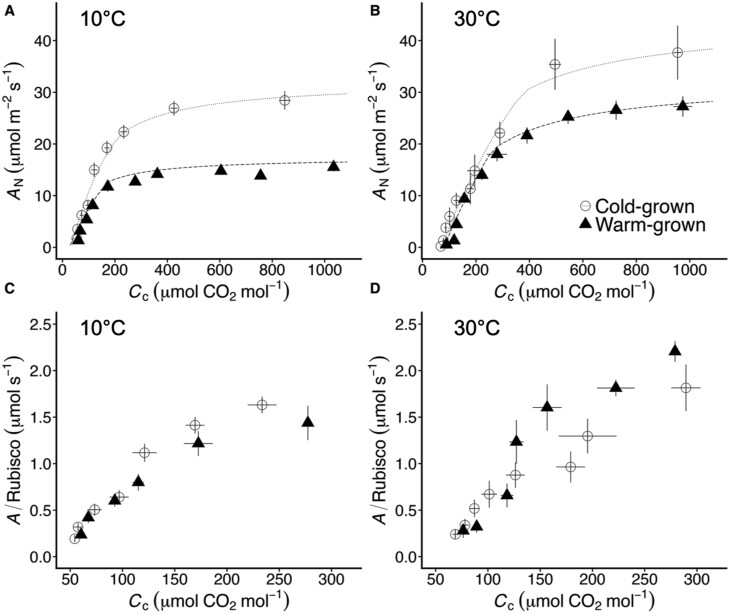
Photosynthetic carbon assimilation in warm- and cold-grown plants. (A, B) Measured (symbols) and modelled (lines) *A*–*C*_c_ responses of cold-grown (open circles) and warm-grown (triangles) plants at 10 °C (A) and 30 °C (B). The modelled response uses the Rubisco parameters and activation energies reported in Table 2 in the Farquhar *et al.* (1980) model. Other modelling parameters are shown in Table 1. Mesophyll conductance to CO_2_ (*g*_m_) was estimated through curve fitting, following Ethier and Livingstone (2004) (see ‘Materials and methods’). (C, D) Assimilation per Rubisco concentration at 10 °C (C) and 30 °C (D) as (*A*_N_+*R*_d_)/Rubisco concentration and illustrates changes in Rubisco biochemistry *in vivo* in the Rubisco-limited portion of the *A*_N_–*C*_c_ response in (A, B). Data represent means ±SE of 4–7 replicates.

Estimated from the initial slope of the *A*_N_–*C*_c_ curve *in vivo*, the Rubisco Michaelis–Menten constant under 21% oxygen (*K*_C21%O2_) increased with measurement temperature (Table 3), but did not differ with growth temperature when measured at 10 °C. By contrast, cold-grown Arabidopsis plants had a 37% higher *K*_C21%O2_ than warm-grown plants when measured at 30 °C (*P*<0.05; Table 3). Consistent with these observations, the initial slope of the photosynthetic CO_2_ response curve was lower in warm- than cold-grown plants measured at 10 °C, but not at 30 °C where warm-grown plants could maintain the same initial slope as cold-grown plants (Fig. 4B). Warm-grown plants had lower photosynthetic rates than cold-grown plants at both measurement temperatures, but the effect was less pronounced at 30 °C. When measured at ambient CO_2_ (corresponding to *C*_a_=400 µmol mol^−1^ CO_2_, or *C*_c_~170–250 µmol mol^−1^ CO_2_), net CO_2_ assimilation (*A*_N_) in cold-grown plants was 62% greater than in warm-grown plants at 10 °C, but did not differ from the warm-grown rate of net assimilation at 30 °C (Fig. 4; Table 1). At 30 °C, warm-grown plants maintained higher rates of assimilation on a Rubisco basis, while at 10 °C assimilation rates per Rubisco did not differ between warm- and cold-grown plants (Fig. 4C, D).

## Discussion

In this study, we investigated the impact of differential expression of Rubisco small subunit isoforms on carbon assimilation in Arabidopsis. Although previous reports have demonstrated that gene specific expression of the *RbcS* family varies with growth condition, impacts on Rubisco catalytic performance or carbon assimilation remain unclear. Here, we demonstrate that in Arabidopsis Rubisco small subunit gene expression and protein production change with growth temperature such that warm-grown plants produce Rubisco containing ~65% SSu-B and cold-grown plants produce Rubisco with ~65% SSu-A as a proportion of the total pool of subunits (Figs 1, 2). These structural changes are accompanied by modifications to Rubisco’s performance *in vitro*; warm-grown plants produce a Rubisco having greater CO_2_ affinity (i.e. higher *S*_C/O_ and lower *K*_C_) but lower *k*_catCO2_ at measurement temperatures above 25 °C (Table 2; Fig. 3). Changes in the kinetic phenotype of Rubisco are also evident *in vivo*, with warm-grown Rubisco having a lower *K*_C21%O2_ than cold-grown Rubisco at 30 °C (Table 3). At 30 °C, warm-grown plants maintain assimilation rates similar to cold-grown plants at ambient CO_2_ concentrations, despite the latter having 60% more Rubisco on a leaf area basis, indicating that the carboxylation capacity of warm-grown Rubisco is enhanced at warmer measurement temperatures, and is able to compensate for the lower Rubisco content in warm-grown plants (Fig. 4; Table 3). These findings provide insight into Rubisco’s performance in a variable climate, which will have increasing importance in a rapidly warming world.

### Rubisco SSu isoform expression varies with growth temperature in Arabidopsis

Many photosynthetic proteins exist in multigene families, in which the expression of certain genes is up- or down-regulated in response to changes in the growth environment (Simpson *et al.*, 1986; Green *et al.*, 1991). In Arabidopsis, Rubisco gene expression has been reported to respond to changes in light, CO_2_, and growth temperature (Dedonder *et al.*, 1993; Cheng *et al.*, 1998; Yoon *et al.*, 2001; Sawchuk *et al.*, 2008). Typically, these responses involve changes to the total amount of *RbcS* mRNA produced, which correlate well with Rubisco production in many species (Moore *et al.*, 1999). Here, we found that in warm-grown plants, the total *RbcS* transcript abundance decreases by 26% compared with cold-grown plants, and this is coupled with a 38% decrease in Rubisco content (Figs 1, 2), supporting the suggestion that the changes in *RbcS* expression facilitate a coarse control over Rubisco enzyme production (Ogawa *et al.*, 2012). However, Rubisco production involves a number of processes, including transcription, translation and/or post-translation (e.g. protein turnover) events, and availability of the SSu (Berry *et al.*, 1985, 1986; Deng and Gruissem, 1987; Shirley and Meagher, 1990; Suzuki and Makino, 2012; Hanson *et al.*, 2021). Recent work demonstrated that the expression of both *rbcL* and *RbcS* did not match the diel pattern of Rubisco protein abundance in wheat, supporting the importance of post-transcriptional regulation in Rubisco production (Perdomo *et al.*, 2021). Supporting this, we found that cold-grown Arabidopsis plants produce more Rubisco than 20 °C-grown plants (Table 1), but the total *RbcS* mRNA does not increase accordingly (Fig. 1). Additionally, we found that Arabidopsis gene specific *RbcS* expression is coordinated with production of the corresponding SSu peptides in the Rubisco holoenzyme (Figs 1, 2). In Arabidopsis, it seems varied *RbcS* expression has a function beyond the control of Rubisco content.

### Arabidopsis Rubisco kinetics vary in response to growth temperature

Rubisco temperature responses across lineages have now been widely characterized, revealing natural variation. Despite general trends for increasing *k*_catCO2_ and decreasing *S*_C/O_ with increasing temperature, variation in Rubisco temperature responses exist even among closely related species (Galmés *et al.*, 2015, 2016, 2019; Perdomo *et al.*, 2015; Orr *et al.*, 2016; Sharwood *et al.*, 2016). Early data suggested that environmental factors, such as temperature and availability of CO_2_ and O_2_, may have selected for ‘better’ versions of plant Rubiscos whose performance was well adapted to local climate. For example, C_3_ species from cool habitats have been found to have an enhanced *k*_catCO2_ with lower activation energy than warm-native C_3_ species, while Mediterranean species native to hot and dry conditions have an increased *S*_C/O_ (Sage, 2002; Galmés *et al.*, 2005). However, in a meta-analysis of 138 species, incorporating recent large-scale screening of Rubisco temperature responses, these comparisons were found to be non-significant, suggesting limited adaptive changes in Rubisco (Galmés *et al.*, 2019). Further, Orr *et al.* (2016) found that Rubisco from warm temperature environments had increased oxygenation rates and affinity for O_2_, resulting in a negative correlation between *S*_C/O_ and warm temperature environments. Although these results may be impacted by a sampling bias for crop species, or variation in temperature ranges and assay method, potential plasticity in Rubisco temperature response could also have an impact on large-scale screening results. Large multi-species screens, such as that by Orr *et al.* (2016) and Hermida-Carrera *et al.* (2016) represent a common garden experiment, whereby the impacts of growth temperature and climate of origin on Rubisco performance cannot be fully separated.

Here we found that cold-grown Arabidopsis produces Rubisco with a higher *k*_catCO2_ than warm-grown plants at all measurement temperatures (Fig. 3A; Table 2). The activation energy of *k*_catCO2_ did not vary with growth temperature, which is consistent with the lack of ecological adaptation noted above. Warm- and cool-grown plants have a similar *S*_C/O_ at 25 °C, but differences in the apparent activation energy result in warm-grown Rubisco being more specific than cold-grown Rubisco at measurement temperatures above 25 °C (Fig. 3B). This is similar to the acclimation response previously observed in spinach Rubisco (Yamori *et al.*, 2006). The observed changes in Arabidopsis *S*_C/O_ are likely related to changes in *K*_c_ between cold- and warm-grown Rubisco; the increased activation energy of this parameter in cold-grown plants results in a *K*_c_ that is 26% greater at 35 °C than that of the warm-grown Rubisco (Table 2; Fig. 3C). Similarly, at low measurement temperatures (<10 °C), cold-hardened *Secale cerale* (winter rye) Rubisco *K*_c_ is 50% lower than non-hardened (25 °C grown) Rubisco; measured above 25 °C, *K*_c_ of the cold-hardened enzyme is double that of non-cold-hardened Rubisco (Huner and Macdowall, 1979). It is not clear why low growth temperatures do not confer a biochemical advantage to Rubisco at low measurement temperatures in Arabidopsis, though it may be that increased Rubisco production (Table 1) compensates for any selection pressure for improvement (Fig. 4A, C).

### Relationship between SSu isoform abundance and Rubisco performance

Differential expression within multigene families can allow for flexible responses to diverse environmental signals. In barley, the alcohol dehydrogenase (ADH) gene family results in six different ADH isoforms (Hanson and Brown, 1984); the concentration of each isoform responds differently to oxygen levels (Hanson *et al.*, 1984). In maize variable LHCII isoforms accumulate to different levels depending on growth temperature and light conditions; some isoforms increase non-photochemical quenching, suggesting a specific role for variable LHCII complexes (Caffarri *et al.*, 2005). In some species, different isoforms of Rubisco activase are produced at elevated growth temperature, with different rates of ATPase or Rubisco activation, and can impact Rubisco activity and photosynthesis under these conditions (Crafts-Brandner *et al.*, 1997; Law and Crafts-Brandner, 2001; Degen *et al.*, 2020, 2021; Kim *et al.*, 2021). Here, we found that warm-grown Arabidopsis produces more of the SSu-B isoform, and less of SSu-1A, than do cold-grown plants, and the corresponding Rubiscos are more specific for CO_2_ versus O_2_ at elevated measurement temperatures. This suggests that differential small subunit expression could contribute to plasticity in enzyme function and activity, and may confer on a plant the ability to fine tune Rubisco through the expression of variable SSu isoforms.

We found differences in *k*_catCO2_ between plants from contrasting growth temperatures at 25 °C, but differences in *K*_C_ and *S*_C/O_ were only apparent at measurement temperatures above 35 °C, highlighting the need to measure Rubisco kinetics under more than one measurement temperature. Previous work has also demonstrated that mutations in Arabidopsis Rubisco SSu isoforms do not impact photosynthesis or holoenzyme kinetic properties under the present atmospheric CO_2_ concentrations at measurement temperatures of 25 °C (Izumi *et al.*, 2012; Atkinson *et al.*, 2017). Lin *et al.* (2020) investigated the impact of homogeneous SSu composition in recombinant tobacco Rubisco and found no differences in Rubisco kinetics at 25 °C attributed to subunit composition beyond the increased *k*_catCO2_ and lower *K*_C_ conferred by the distinct trichome SSu (Laterre *et al.*, 2017; Lin *et al.*, 2020). This supports earlier attempts to mix and match tobacco Rubisco subunits via interspecific hybridizations, which also found no impact of SSu composition on Rubisco activity (Li *et al.*, 1983). However, the kinetics of potato Rubisco expressed in tobacco were significantly affected by the identity of the SSu (Martin-Avila *et al.*, 2020), and a recent survey of recombinant Solanaceae ancestral Rubisco suggests that the SSu influences the kinetic phenotype of the enzyme, in particular at warmer temperatures (Lin *et al.*, 2022). The minor differences in the Arabidopsis SSu polypeptides are found in regions known to influence Rubisco kinetics in other organisms (Valegard *et al.*, 2018) and may play a role in holoenzyme stability or CO_2_ affinity (van Lun *et al.*, 2014; Poudel *et al.*, 2020), which would have a pronounced impact at elevated temperatures.

### Plant acclimation to growth temperature: Rubisco responses *in vivo*

We demonstrated that growth temperature alters Arabidopsis Rubisco kinetic performance *in vitro* and growth-temperature induced changes in Rubisco biochemistry are also evident *in vivo*, with warm-grown Rubisco having a lower apparent *K*_C21%O2_ than cold-grown Rubisco at 30 °C (Table 3). Additionally, there is no difference in the initial slope of the photosynthetic CO_2_ response between warm- and cold-grown plants at 30 °C, indicating that the enhanced carboxylation capacity of warm-grown Rubisco compensates for the lower Rubisco content in warm-grown plants (Fig. 4A, B; Table 3). As a result, warm-grown plants maintain rates of CO_2_ assimilation (*A*) similar to cold-grown plants at 30 °C and ambient (or lower) CO_2_ concentrations, despite their reduced Rubisco content (Fig. 4C, D). This ability could provide a photosynthetic advantage during times of stomatal closure, such as drought conditions, which are frequently associated with growth at elevated temperatures in natural and agricultural systems.

**Fig. 5.**
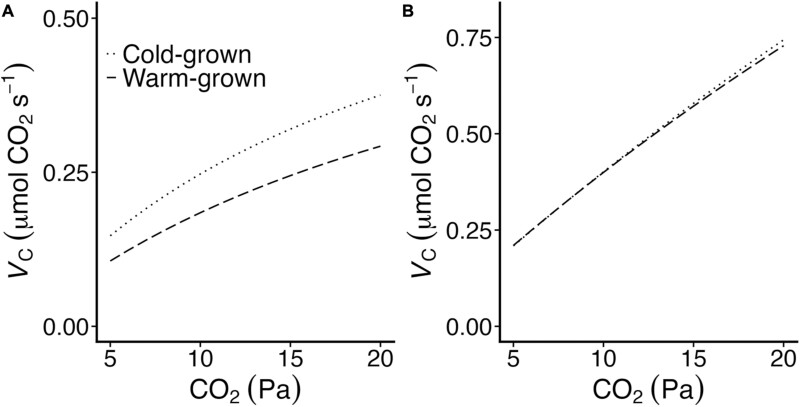
Modelled responses of Rubisco carboxylation accounting for differences in growth temperature changes to kinetics. The impact of changes in Rubisco *K*_C_ (Table 2) are modelled for cold-grown (dotted line) and warm-grown (dashed line) Rubisco at 10 °C (A) and 30 °C (B).

In spinach, similar changes in Rubisco performance offer a theoretical carbon assimilation advantage to a warm-grown plant (Cavanagh and Kubien, 2014). However, when changes in spinach Rubisco *S*_C/O_ are driven by differences in *K*_C_, as they appear to be in Arabidopsis (Table 2; Fig. 3), a carbon gain advantage for a warm-grown plant is only found at temperatures greater than approximately 32–33 °C (i.e. at temperatures above the growth temperature). When the observed differences in *K*_C_ are used to model the rate of Rubisco carboxylation on an equal enzyme concentration basis (i.e. *V*_C_), the initial slopes of the CO_2_ response are similar at 30 °C, but not 10 °C (Fig. 5), similar to the results of our *in vivo* CO_2_ response curve. However, at ambient and higher CO_2_ concentrations, the lower photosynthetic rate in warm-grown plants is a likely result of strict limitations imposed by decreased Rubisco content; warm-grown plants produce 38% less Rubisco than cold-grown plants (Table 1). Warm-grown plants do maintain rates of CO_2_ assimilation similar to cold-grown plants at 30 °C and at ambient and sub-ambient CO_2_ concentration (Fig. 4B), which is likely a reflection of their lower *K*_C21%O2_ and higher *S*_C/O_ at this temperature.

### Effects of Rubisco phenotypic plasticity on photosynthetic performance and modelling

Plasticity in Rubisco performance may have important implications for modelling photosynthesis, as the CO_2_ assimilation rate of most species is modelled using *in vivo* Rubisco kinetic parameters obtained from tobacco or Arabidopsis, and the effect of growth environment on Rubisco performance or content is not considered (Bernacchi *et al.*, 2001; Walker *et al.*, 2013). Further, *in vivo* Rubisco temperature responses in Arabidopsis and tobacco have been obtained using an antisense knockdown of a single *RbcS* gene, which may bias the temperature response. Despite the lack of species-specific differences in a single Rubisco biochemical parameter between Arabidopsis and tobacco grown at the same temperature, estimates of *V*_cmax_ obtained from a CO_2_ response curve vary depending on the choice of temperature response parameters used, highlighting the importance of small variation in Rubisco parameters for modelling photosynthesis (Walker *et al.*, 2013). The effect of growth temperature-induced changes, or modulations via the SSu, particularly on *S*_C/O_, *K*_C_, and their activation energies (Table 2), could result in species-specific differences between parameters that impact photosynthetic models.

## Supplementary data

The following supplementary data are available at *JXB* online.

Fig. S1. Combinatorial temperature and CO_2_ treatments on *RbcS* expression.

Fig. S2. Response of low abundance *RbcS* isoforms to growth temperature.

Fig. S3. Temperature response of Rubisco *S*_C/O_ above 20 °C.

Table S1. A list of primers used in this work.

erac379_suppl_Supplementary_Table_S1_Figures_S1-S3

## Data Availability

The data are available upon request from the corresponding author (APC).

## Abbreviations:

*A*_N_: net CO_2_ assimilation
*C*_a_: CO_2_ concentration in air
*C*_i_: intercellular airspace CO_2_ concentration
*C*_c_: chloroplastic CO_2_ concentration
*E*_a_: activation energy
*g*_m_: mesophyll conductance to CO_2_
*g*_s_: stomatal conductance to CO_2_
*J*_max_: maximal rate of electron transport
*K*_C_: Michaelis–Menten constant of Rubisco for CO_2_
$K_{\text{C21}\%{\text{O}}_{\text{2}}}$: Michaelis–Menten constant of Rubisco for CO_2_ in the presence of 21% O_2_
$k_{{\text{catCO}}_{\text{2}}}$: catalytic turnover rate of Rubisco carboxylation
*K*_O_: Michaelis–Menten constant of Rubisco for O_2_
LSu: Rubisco large subunit
*rbcL*: gene encoding Rubisco large subunit
*RbcS*: gene encoding Rubisco small subunit
RuBP: ribulose-1,5-bisphosphate
*S*_C/O_: Rubisco substrate specificity
SSu: Rubisco small subunit
Γ*: CO_2_ compensation point
*V*_c_: Rubisco carboxylation rate
*V*_cmax_: maximal rate of carboxylation
