# Supplementary material for: Temperature-induced changes in Arabidopsis Rubisco activity and isoform expression
Source: J Exp Bot. 2022 Sep 17;74(2):651–63. doi: 10.1093/jxb/erac379 (PMC9833042; doi:10.1093/jxb/erac379)
Supplement: erac379_suppl_Supplementary_Table_S1_Figures_S1-S3 [file erac379_suppl_supplementary_table_s1_figures_s1-s3.pdf]

Table S1: A list of primers used in this work. All rbcS primer pairs have a common forward primer (rbcSF).

Gene	Primer Sequence (5'-3')	Tm (°C)
rbcS-F	GGATCATCGGATTCGACAAC	64.2
rbcS1A-R	AATATGTCTCGCAAACCGGA	64.2
rbcS1B-R	GAGGAAACGATAGAAAAACAAACCT	63.2
rbcS2B-R	GAGGATAATTTAAAGGAGCCACAAT	63.0
rbcS3B-R	TAAATCAGACATTTGACAATCCGA	64.1
rbcL-F	GTGTTGGGTTCAAAGCTGGT	63.9
rbcL-R	CATCGGTCCACACAGTTGTC	64.2
AT5G0829-F	ATGAGGTGCTTGCGTCTGTT	63.7
AT5G0829-R	TCCTTGAGAGCCCAGTTGAT	63.7
AT1G13320 F	TAACGTGGCCAAAATGATGC	65.7
AT1G13320R	GTTCTCCACAACCGCTTGGT	66.2

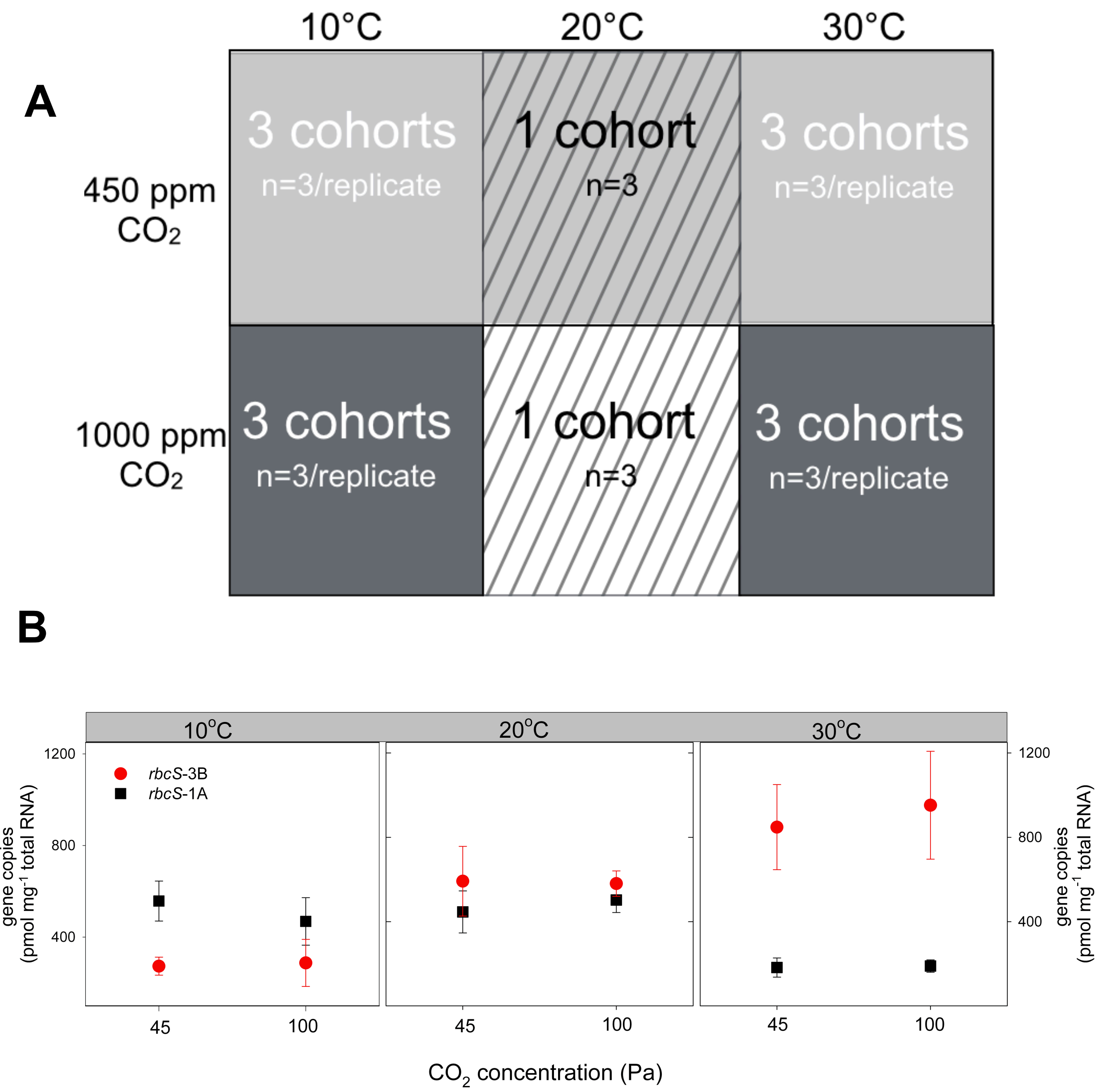

Fig S1: Combinatorial temperature and CO_2 treatments on *rbcS* expression. Experimental design (A) to test combinatorial effects involved three cohorts of plant growth in each condition. Analysis with a linear mixed effects model shows no impact of CO_2 concentration (B) on expression of *rbcS*1A (black squares) or *rbcS*3B (red circles).

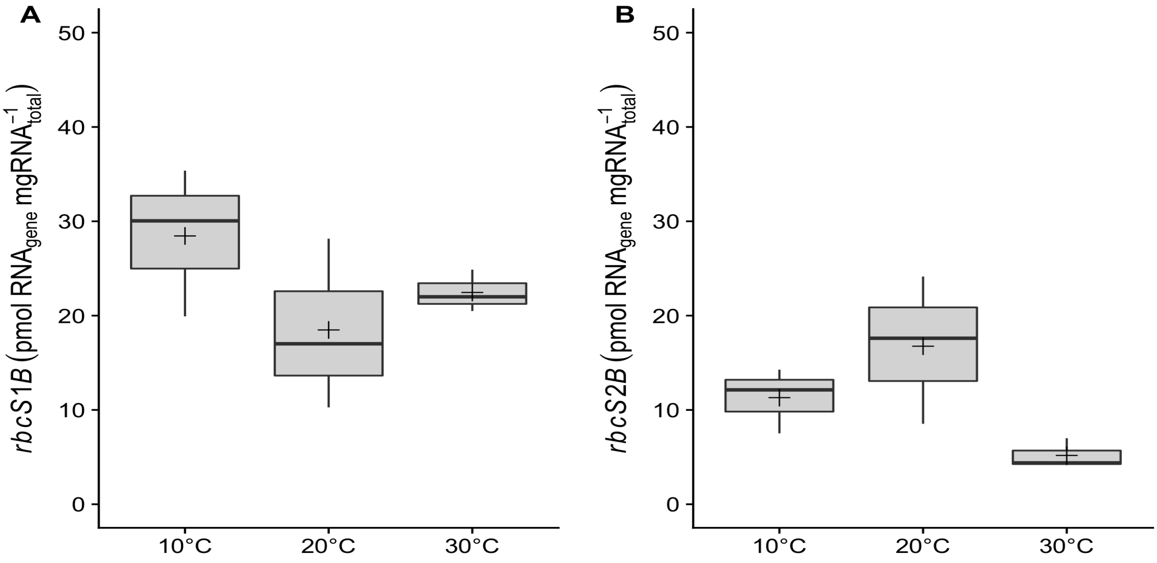

Fig S2: Response of low abundant *rbcS* isoforms to growth temperature. *rbcS*1B (A) and *rbcS*2B (B) do not vary with growth temperature. Gene specific expression is reported as the fraction of total RNA concentration n=6-7. Protein expression is reported as the fraction of total small subunit protein. Maximum and minimum values are depicted by the bars, the box signifies the upper and lower quartiles and the median is represented by a short black line within each box. No differences at any measurement temperature (one-way ANOVA).

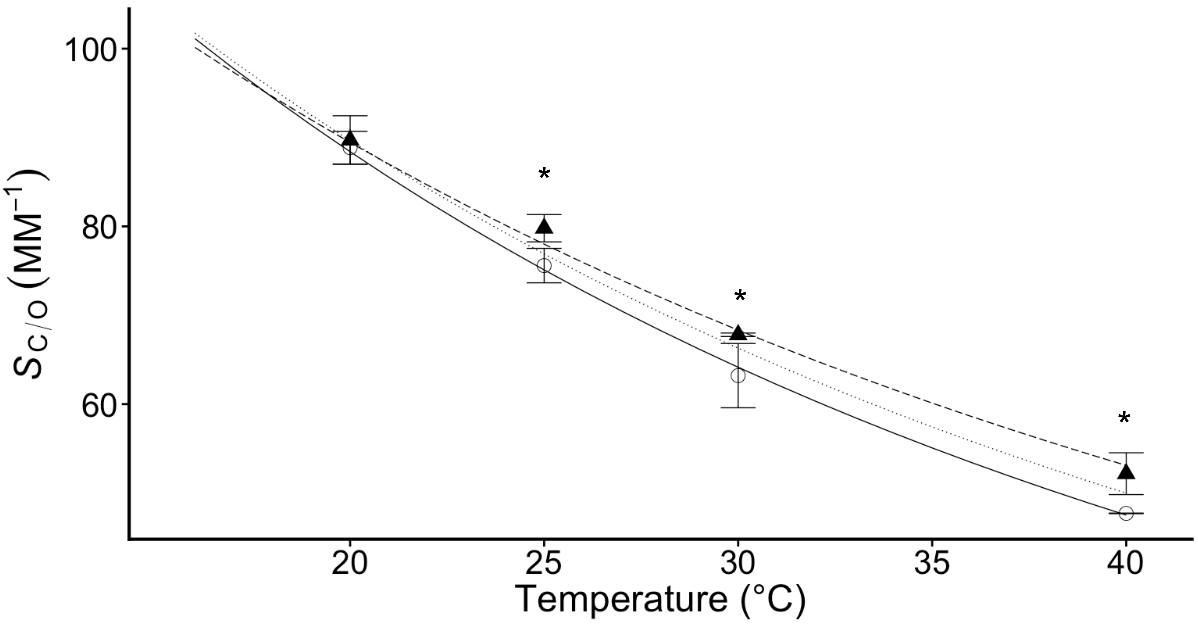

Fig S3: Temperature response of Rubisco $S_{C/O}$ above 20°C. Temperature dependence of the in vitro Rubisco $S_{C/O}$ from warm-grown (triangles) and cold-grown (open circles) Arabidopsis. Parameters were determined by assaying activated Rubisco extracts at the indicated temperatures. Data represent means ± standard error of 4-7 replicates. Lines represent the temperature response of cold (solid) and warm (dashed) Rubiscos, and the dotted line is the temperature response from 20°C grown Arabidopsis Rubisco as reported in Boyd et al., 2019.
